# A Small Number of Gametophytes with Gametangia and Stunted Sporophytes of *Antrophyum obovatum* Baker (Pteridaceae): The Suppression of Functional Sporophyte Production by Prezygotic and Postzygotic Sterility

**DOI:** 10.3390/plants10010170

**Published:** 2021-01-18

**Authors:** Sang Hee Park, Jung Sung Kim, Hyoung Tae Kim

**Affiliations:** 1 Department of Forest Science, Chungbuk National University, Chungdae-ro 1, Chungbuk 28644, Korea; pleura@naver.com; 2 Institute of Agricultural Science and Technology, Chungbuk National University, Chungdae-ro 1, Chungbuk 28644, Korea

**Keywords:** *Antrophyum obovatum*, Pteridaceae, independent gametophytes, asexual reproduction

## Abstract

Ferns have conspicuous sporophytes as the dominant phase in their life cycle; however, the gametophytes are completely separated from the sporophytes and supply their own nutrition, unlike in bryophytes and seed plants. Among the gametophytes, some maintain their populations in the gametophyte phase without progressing to sporophyte production and are known as independent gametophytes. Independent gametophytes of *Antrophyum obovatum* Baker were recently reported in one population on Jeju Island, Korea. In the present study, we surveyed more places to find new independent gametophyte populations of *A. obovatum* using the *rbcL* gene sequence-based DNA barcoding technique. We identified two new sites inhabited by independent gametophytes. Archegonia and juvenile sporophytes were independently observed in each location under slightly different environmental conditions. Consequently, in the case of this species, functional sporophyte production is likely suppressed by prezygotic and postzygotic sterility, depending on microenvironmental factors.

## 1. Introduction

All land plants generally have distinct gametophyte and sporophyte phases. In the life cycle of bryophytes, the gametophyte (as the sexual phase) is dominant in the alternation of generations, and the sporophyte (as the asexual phase) is attached to the gametophyte like an appendage. However, this has been altered in seed plants, and the gametophytes are less developed than the sporophytes and grow entirely inside the sporophyte. In the case of ferns, which belong to vascular plants but are not seed plants, there are also conspicuous sporophytes as the dominant phase (like in seed plants); however, they differ in that the gametophytes are completely separated from the sporophytes and supply their own nutrition.

Nayar and Kaur [1] classified fern gametophytes into five types based on their morphological characteristics: tuberous, filamentous, cordate-thalloid, strap-like, and ribbon-like. Most ferns have cordate-thalloid gametophytes, grow quickly, and live for less than one year in the natural environment, with the exception of some lineages [1,2,3]. These gametophytes are developed by a single apical cell meristem in the notch at the apex of the thallus, followed by a multicellular meristem and the immediate production of an archegonial cushion [2]. However, noncordate gametophytes, especially epiphytic and epipetric species, generally grow slower and live longer than cordate gametophytes [1,3,4]. Some of these produce gemmae or strap-shaped lobes for asexual reproduction [5,6,7], and a few gametophytes maintain their populations and are known as independent gametophytes [8]. Independent gametophytes have occasionally been reported in four families: Hymenophyllaceae, Lomariopsidaceae, Polypodiaceae, and Pteridaceae [9,10,11,12,13,14,15,16]. Rumsey and Sheffield [17] distinguished obligate independent gametophytes from facultative ones depending on the existence of conspecific functional sporophytes in nature; however, Kuo, Chen, Shinohara, Ebihara, Kudoh, Sato, Huang, and Chiou [14] proposed a new definition for identifying fern gametophyte independence at the population level (Table 1). We followed this concept.

Independent gametophytes have mainly been discovered in temperate and continental regions [10,11,13,18,19,20,21], whereas sporophytes producing gemmiferous gametophytes have highly diverged in tropical and subtropical regions and islands [18]. Recently reported independent gametophytes in subtropical regions indicated that recent climate change during the Pleistocene glaciation led to prezygotic sterility and that gametophytes have maintained their populations by producing gemmae [14].

*Antrophyum* Kaulf is a member of the vittarioid ferns and comprises about 40 to 50 unclear species that are widespread in the tropics of both the Old and New World [22]. The morphology of the gametophytes of this family is characterized by a branched ribbon-like prothallus with short filamentous gemmae [1]. *Antrophyum obovatum* Baker is mainly distributed in the tropical or subtropical areas of Asia, including the South Yangzi River in China, Taiwan, Vietnam, Thailand, Myanmar, India, Bhutan, Nepal, and the southern area of central Japan [22]. The gametophyte of *A. obovatum* was first reported on Jeju Island in Korea, and our previous research suggested that it was an obligate independent gametophyte based on molecular markers [23]. However, in this situation, only a very small number of individuals were studied in one population and no gametangia remained, which raised the following questions: (1) Are the gametophytes of *A. obovatum* extremely rare? (2) Is prezygotic sterility the only reason for the change from general gametophytes to independent gametophytes? (3) Are conspecific sporophytes absent on Jeju Island? Therefore, we surveyed more places to find new populations of this species on Jeju Island and carefully observed them to address these questions in detail.

As fern gametophytes are relatively simple and not differentiated into specialized organs compared with sporophytes [1], it is very difficult to identify gametophytes at the species level based on their morphological characteristics. Therefore, DNA barcoding techniques have been widely applied to identify fern gametophytes at the species level [15,16,24,25]. Although the Consortium for the Barcode of Life proposed *rbcL* and *matK* for plant DNA barcode regions in terms of high universality and high variation, respectively [26], the *rbcL* region also has high variation in ferns to identify nonhybridized gametophytes [27] and has been sequenced from most fern families [27,28,29,30], including Vittariaceae [31,32]. For these reasons, we used *rbcL* regions to identify gametophytes and juvenile sporophytes in this study.

## 2. Results

### 2.1. Identification Based on the rbcL Sequence

Polymerase chain reaction (PCR) amplification of *rbcL* was successful in all samples of the 12 gametophytes and sporophytes. There was no variation in the *rbcL* sequences of the gametophytes and sporophytes among the three populations on Jeju Island, and they were completely identical to the *A. obovatum* sporophyte sequences collected from Japan and Taiwan.

### 2.2. Habitat

**Figure 1:**
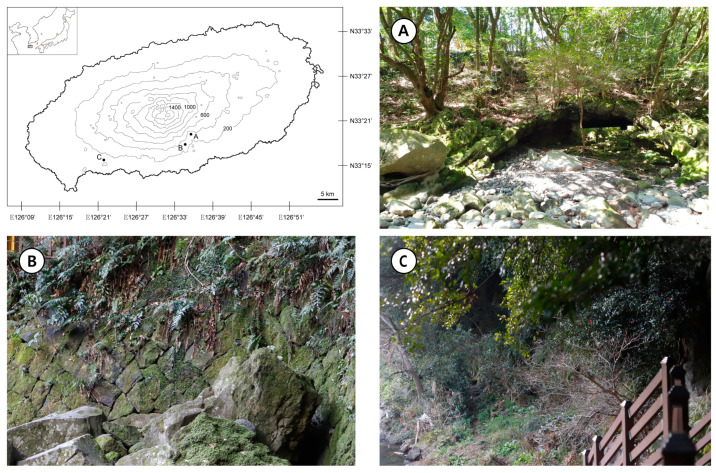
Collection sites. (**A**) Youngcheon-dong, shady bunker in a relatively dry valley. (**B**) Youngcheon-dong, shady and humid valley. (**C**) Andeok-myon, open steep slope under a canopy in a relatively dry valley.

We randomly surveyed a number of valleys to determine the presence of gametophytes and sporophytes of *A. obovatum* because the condition of the first site was incongruent with the locations where the sporophytes of *A. obovatum* inhabit tree trunks or rocks [23,33]. As a result, the gametophytes of *A. obovatum* were newly found at two sites, but the environmental conditions of the sites were slightly different. Site A was a shady bunker built by stacked stones in the upper valley under dry conditions (Figure 1A). A large colony covered the bunker wall. Site B was on the stone wall staircase, 2.7 km away from site A, and was very close to a waterfall and a stream (Figure 1B). Therefore, site B was more humid than site A. A large colony covered the artificial wall. The altitudes of sites A and B were 304 and 290 m, respectively. Site C, which was reported as the first site to contain a population of *A. obovatum* gametophytes, was close to the stream in a valley but was in an open area (Figure 1C). Therefore, it had relatively dry conditions. A small colony grew on a steep soil slope. We tried to find other populations within a 200 m radius from the three sites because sites A and B were artificial structures that were less than hundreds of years old. However, no other population has been discovered to date.

### 2.3. Morphological Characteristics of Gametophytes and the Juvenile Sporophyte

**Figure 2:**
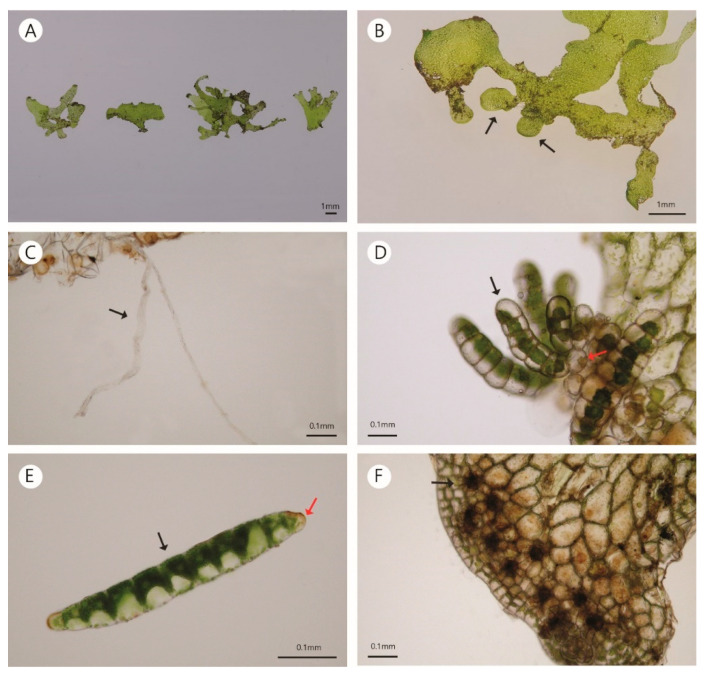
Morphology of the gametophytes of *Antrophyum obovatum*. (**A**) Various forms of the thallus. (**B**) Irregular branching (black arrow). (**C**) Rhizoid. (**D**) Gemma (black arrow) and gemmifer (red arrow) at the end of aerial branches. (**E**) Body cells (black arrow) and rhizoid primordia (red arrow) of the gemma. (**F**) Archegonium (black arrow) at the marginal lobes of horizontal branches.

The thallus of the gametophyte was ribbon-like and consisted of a single cell layer (Figure 2A). The branching pattern was irregular because of the discontinuous marginal meristem (Figure 2B). Rhizoids mainly arose from the basal margin that came in contact with the substrata or occasionally from inner cells, with a whitish brown color (Figure 2C). The ends of the aerial branches of the gametophytes had several gemmifers, and one to three gemmae were attached to a gemmifer (Figure 2D). The mature gemma was uniseriate with a spindle form consisting of 8–14 cells, and the rhizoid primordia cells were positioned at both ends without chloroplasts (Figure 2E). Archegonia were not found at sites A and C when we investigated 50 individuals. However, they were occasionally found in six of the 30 individuals examined at site B (Figure 2F). Antheridia were not found in any of the populations.

**Figure 3:**
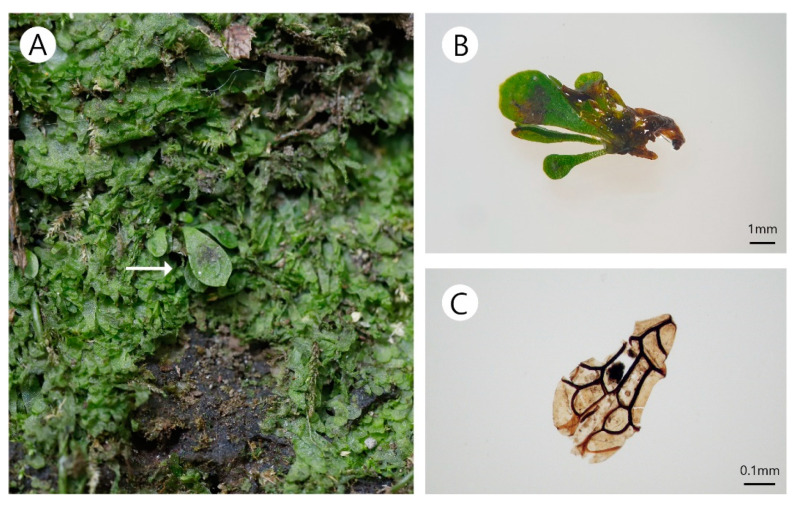
Morphology of the juvenile sporophyte of *Antrophyum obovatum*. (**A**) Habitat of sporophytes. (**B**) Juvenile sporophyte. (**C**) Scale of rhizome.

A few juvenile sporophytes grew within the patch of gametophytes at site B (Figure 3A). The leaves were glabrous, obovate, round at the apex, and less than 6 mm in length (Figure 3B). The rhizomes were short and erected. The rhizome scales were dark brown, ovate, lanceolate, and clathrate (Figure 3C).

The gametophytes remained in the laboratory for more than two years after collection; however, gemmae did not arise from any individual. The juvenile sporophytes wilted six months after translocation, without any morphological changes.

## 3. Discussion

For two years, we surveyed several valleys on Jeju Island to identify new populations of independent gametophytes of *A. obovatum*, but new populations were only discovered at two sites that were close to each other. Because it was impossible to exhaustively search all valleys on Jeju Island and because independent gametophytes of *A. obovatum* were too small to find easily in nature, we are not sure whether they are rare or overlooked. However, it is clear that the populations of sites A and B came from other populations because both sites were artificial structures. Although we could not find additional populations within a 200 m radius of sites A and B after exhaustive investigation, independent gametophytes of *A. obovatum* completely covered the stone walls of sites A and B. It is likely that the condition for the establishment of a gametophyte population was very limited, depending on the microhabitat, and that one or more gametophytes rapidly proliferated due to no competition under asexual reproduction before exceeding the capacity of the newly colonized environment, such as in the founder-flush speciation theory [34].

### 3.1. Suppression of Archegonia and Gemmae by Micro-Environmental Factors

Before discussing the archegonia and gemmae on the gametophytes of *A. obovatum*, we must first briefly discuss the absence of antheridia in all of the investigated populations. In vittarioids, the antheridia generally arise from very young plants and germinating gemmae, with the exception of *Ananthacorus*, in which archegonium-bearing gametophytes have scattered antheridia [35]. Based on the presence of juvenile sporophytes, it could be assumed that the antheridia were at least produced in the site B population. Because the antheridia may easily come out from small gametophytes and gemmae when they were cleaned for the microscopic study [36] and occur much less than archegonia in the other independent gametophytes of *Pleurosoriopsis makinoi* [21], the absence of antheridia does not mean there was a malfunction of antheridium formation in the independent gametophytes. More detailed information from a long-term investigation of antheridium formation dependent on ecological conditions is necessary to understand the antheridium formation of *A. obovatum* gametophytes.

From our observations, there were two differences between the gametophytes of *A. obovatum* that were dependent on the environment. First, the producing gemmae were suspended in individuals translocated in the laboratory. Translocated individuals used in the previous study [23], as well as those sampled for this study, are still alive with meristematic activity, but there have not been new gemmae from the thalli. Although the experimental conditions were not identical, it was reported that the gametophytes of other *Antrophyum* species germinated from spores produced gemmae on the media in the laboratory [31], which was also true for other vittarioids [35]. It is possible that the absence of nutrients, which seems to be the main difference between the present growing conditions and previous ones, stopped the production of gemmae; however, the survival period and meristematic activity of the gametophytes for more than two years in the present study implied that they could produce their own food using photosynthesis. Second, archegonia were only found at site B under humid conditions. Conversely, gametophytes under dry conditions did not produce female sexual organs. This could be due to seasonality or an alternation between active and inactive years; however, none of the gametophytes observed at site C surveyed from 2018 had archegonia. Consequently, we concluded that there are three types of *A. obovatum* gametophytes: (1) nongemmiferous gametophytes without archegonia (under laboratory conditions), (2) gemmiferous gametophytes without archegonia (sites A and C), and (3) gemmiferous gametophytes with archegonia (site B). What makes these differences?

Based on the observations of independent gametophytes of *P. makinoi* [21], there were clear seasonal patterns for gemmae formation and growth, with the highest number of gemmae observed from late summer to autumn and the extension of their size before separation from the thalli. This gemma production is similar to the flowering pattern in angiosperms. In most flowering plants, flowers only appear at certain times of the year, with the coordination of the onset of flowering by environmental cues [37]. As a result, climate change influences the flowering date of flowering plants [38]. Therefore, if the independent gametophytes also have control switches for gemma formation, the seasonless condition of the gametophytes in the laboratory might turn off the switches to avoid producing gemmae. However, as the present study was only conducted over the short term and the results are inconsistent with those of other studies (where the gemmae-producing gametophytes of vittarioids germinated from spores in the laboratory [31,35]), this hypothesis must be considered with caution. To verify this hypothesis, gemmaless gametophytes will be returned to their original habitats, and we will investigate whether gemmae are reproduced.

Certain independent gametophytes have reproductive dysfunction that causes the inability to produce sporophytes, which might result from environmental or genetic factors [14,17]. Because gametophytes have better tolerance to cold [39] and dry conditions [40] than conspecific sporophytes, independent gametophytes generally have an extended geographic range beyond that of conspecific sporophytes [12,13,20,23,41,42]. This implies that independent gametophytes have efficiently adapted to new environments and may have a function to regulate the production of gametangia, specifically when it is not beneficial for independent gametophytes (which do not need zygotes) to sustain the population in hostile conditions by developing into sporophytes.

Among the three sites in which the gametophytes of *A. obovatum* reside, site B is more humid than the others because it is surrounded by evergreen trees and has a permanent stream. Humidity affects the number and position of the archegonia. The total number of archegonia in *Pteridium aquilinum* cultured in high humidity (100%) was nearly four-fold higher than that in low humidity (90%), and the anomalous production of archegonia on the dorsal surface, which are normally produced on the ventral surface in nature, significantly appeared in high humidity conditions [3]. Although this experiment cannot provide direct evidence of the absence of archegonia in dry conditions, it implies that humidity is one of the main factors regulating the formation of archegonia. In addition, *Haplopteris flexuosa* (Fee) E. H. Crane was observed at site B, and this is the only vittarioid sporophyte residing on Jeju Island. Our new findings of archegonia and juvenile sporophytes of *A. obovatum* were restricted to only one site, which is consistent with Kuo, Chen, Shinohara, Ebihara, Kudoh, Sato, Huang, and Chiou [14]. When the independent gametophytes of *A. obovatum* at site B encounter a suitable microhabitat for fertilization, they might produce gametangia. As a result, juvenile sporophytes were observed only at site B.

### 3.2. Suppression of Sporophyte Growth by Environmental Factors

In our previous study [23], the gametophytes of *A. obovatum* were considered obligate gametophytes because they were first found in site C and the conspecific sporophytes of these had never been reported in Korea until that time. We wondered if they could not produce gametangia genetically or if the conspecific sporophytes did not actually occur on Jeju Island because no sexual organs were found in the individuals of site C. Fortunately, through successive investigation, we found two additional populations, one of which had archegonia-bearing individuals and then had juvenile sporophytes. Therefore, it was proved that the independent gametophytes of *A. obovatum* do not completely lose the ability to genetically produce gametangia.

Juvenile sporophyte mortality associated with independent gametophytes has been previously reported. In the case of *Vittaria appalachiana*, classified as an obligate independent gametophyte by Rumsey and Sheffield [17], young sporophytes were found in the field and grew to less than 1 cm in length, but they died before producing vascular tissue [36]. The juvenile sporophytes of *Trichomanes speciosum* with independent gametophytes were classified as a facultative independent gametophyte by Rumsey and Sheffield [17] and as an obligate independent gametophyte in certain populations by Kuo, Chen, Shinohara, Ebihara, Kudoh, Sato, Huang, and Chiou [14], and they died following a protracted dry spell [17]. The juvenile sporophytes of *A. obovatum* found in the present study were stunted in nature, and those translocated to the laboratory blanched and died after six months without any morphological changes.

As mentioned above, the three sites inhabited by independent gametophytes had slightly different environmental conditions. Site A was the outside wall of the bunker in a dry valley and was exposed to the sun for a couple of hours per day, while Site C was under shrubs located at the end of a valley connected with flatland. Both sites had higher light levels and relatively lower air humidity than site B, which was located in a valley with a stream and surrounded by evergreen trees. In addition, observations of *H. flexuosa* and archegonia at site B implied that this site is the most suitable place to grow vittarioid ferns compared with other sites on Jeju Island. Independent gametophytes at site B can produce gametangia, and fertilization rarely occurs in this population. However, environmental factors, such as subzero temperatures in winter, are not sufficient for sporophyte survival and might stunt young sporophytes. As a result, sporophytes might die before maturation, and this postzygotic sterility might keep the population as obligate independent gametophytes.

## 4. Materials and Methods

### 4.1. Surveys and Sampling

We surveyed the valleys in southern Jeju Island several times during 2019 and 2020. As a result, we found two new gametophyte populations in Youngcheon-dong (Figure 1A,B) and Andeok-myon (Figure 1C), where gametophytes of *A. obovatum* were first found [23]. During the survey, we found only stunted juveniles in the population at Youngcheon-dong (Figure 1B). To observe their growth pattern, a few gametophytes and sporophytes were collected and transferred to the laboratory to avoid destroying the natural habitat. We simply put these on filter paper with sterile water in a petri dish. We did not apply a medium-based breeding method because the sterilization conditions for the plants were unknown, and some sterilants seemed to weaken or destroy them.

### 4.2. Observation of Morphological Characters

To observe their morphological characteristics, the gametophytes were washed with distilled water and mounted on slides with Entellan^®^ New (Merck, Darmstadt, Germany). Voucher gametophyte and sporophyte specimens were deposited in the herbarium of Chungbuk National University and observed under an Olympus BX50 microscope (Olympus, Tokyo, Japan).

### 4.3. DNA Extraction and PCR Amplification of the rbcL Region for Molecular Identification

Four thalli of the gametophytes from each population and one sporophyte from population B were washed using sterile distilled water several times to remove other bryophytes or soil particles. Each thallus and sporophyte was ground using a homogenizer for three cycles for 30 s at a speed of 3 m/s. DNA was extracted by following the manufacturer’s protocol for the DNeasy Plant Mini Kit (Qiagen, Hilden, Germany). The PCR amplification of the rbcL region for 12 gametophytes and one sporophyte was performed with AccUPower^®^PCR Premix (Bioneer, Daejeon, Korea) with 1 μM of each primer, 1 μM of DNA, and 18 μM of distilled water using Pteridaceae_rbcLF and Pteridaceae_rbcLR designed by Park, Kim, and Kim [23]. PCR conditions were as follows: 5 min denaturation step at 95 °C, followed by 35 cycles at 95 °C for 45 s, 50 °C for 45 s, and 72 °C for 75 s, then followed by a 5 min final extension step at 72 °C. PCR products were purified using a PCR purification kit (Geneall, Seoul, Korea) and sequenced using an ABI 3730×l System (Macrogen, Seoul, Korea).

Voucher specimens were deposited in the herbarium of Chungbuk National University: Gametophyte: Andeok-myon, Seogwipo-si, Jeju-do, Korea. alt. 128 m, 5 December 2018, H.T. Kim, K. Lee & S.H. Park. CBNU2018-0387; Youngcheon-dong, Seogwipo-si, Jeju-do, Korea 298 m, 18 January 2020, S.H. Park. CBNU2020-0001; Youngcheon-dong, Seogwipo-si, Jeju-do, Korea alt. 304 m, 12 March 2020, H.T. Kim & S.H. Park. CBNU2020-0058; sporophyte: Youngcheon-dong, Seogwipo-si, Jeju-do, KOREA alt. 298 m, 18 January 2020, S.H. Park. CBNU2020-0002.

## Figures and Tables

**Table 1:** Classification of fern gametophyte independence.

		Rumsey and Sheffield (1996)	Kuo et al., (2017)
**Precondition**		Gametophyte population maintained only by asexual reproduction	
**Type**		Species level	Population level
**Obligate**	Definition	Absence of conspecific functional sporophytes in nature	Absence of conspecific functional sporophytes in the geographic range of gametophytes
	Example	Only three species: *Vittaria appalachiana* Farrar & Mickel *Crepidomanes intricatum* (Farrar) Ebihara & Weakley *Hymenophyllum tayloriae* Farrar & Raine	Including Rumsey and Sheffield’s obligate gametophytes *Hymenophyllum wrightii* Bosch (Duffy et al., 2015) *Haplopteris* sp. (Kuo et al., 2017)
**Facultative**	Definition	Presence of conspecific functional sporophytes out of the geographic range of gametophytes	Presence of conspecific functional sporophytes in the geographic range of gametophytes but not in touch
	Example	Independent gametophytes except three obligate ones	*Loxogramme grammitoides* (Baker) C. Chr. (Park et al., 2020) *Pleurosoriopsis makinoi* (Maxim.) Fomin (Ebihara et al., 2019)

## Data Availability

Accession numbers deposited in GenBank: MW411044; MW411045; MW411046; MW411047 (Gametophytes in Site A)/MW411040; MW411041; MW411042; MW411043 (Gametophytes in Site B)/MW411036; MW411037; MW411038; MW411039 (Gametophytes in Site C)/MW411048; MW411049 (Sporophytes in Site B).
